# Dimensionality reduction for classification of object weight from electromyography

**DOI:** 10.1371/journal.pone.0255926

**Published:** 2021-08-16

**Authors:** Elnaz Lashgari, Uri Maoz

**Affiliations:** 1 Schmid College of Science and Technology, Chapman University, Orange, California, United States of America; 2 Institute for Interdisciplinary Brain and Behavioral Sciences, Chapman University, Irvine, California, United States of America; 3 Crean College of Health and Behavioral Sciences, Chapman University, Orange, California, United States of America; 4 Department of Biology and Bioengineering, California Institute of Technology, Los Angeles, California, United States of America; 5 Anderson School of Management University of California Los Angeles, Los Angeles, California, United States of America; University of Essex, UNITED KINGDOM

## Abstract

Electromyography (EMG) is a simple, non-invasive, and cost-effective technology for measuring muscle activity. However, multi-muscle EMG is also a noisy, complex, and high-dimensional signal. It has nevertheless been widely used in a host of human-machine-interface applications (electrical wheelchairs, virtual computer mice, prosthesis, robotic fingers, etc.) and, in particular, to measure the reach-and-grasp motions of the human hand. Here, we developed an automated pipeline to predict object weight in a reach-grasp-lift task from an open dataset, relying only on EMG data. In doing so, we shifted the focus from manual feature-engineering to automated feature-extraction by using pre-processed EMG signals and thus letting the algorithms select the features. We further compared intrinsic EMG features, derived from several dimensionality-reduction methods, and then ran several classification algorithms on these low-dimensional representations. We found that the Laplacian Eigenmap algorithm generally outperformed other dimensionality-reduction methods. What is more, optimal classification accuracy was achieved using a combination of Laplacian Eigenmaps (simple-minded) and k-Nearest Neighbors (88% F1 score for 3-way classification). Our results, using EMG alone, are comparable to other researchers’, who used EMG and EEG together, in the literature. A running-window analysis further suggests that our method captures information in the EMG signal quickly and remains stable throughout the time that subjects grasp and move the object.

## Introduction

The neuromuscular activations associated with the contraction potentials of the skeletal muscles generate electrical fields that can be noninvasively recorded and are termed surface electromyograms (EMG) [1]. EMG signals are non-stationary in nature and are affected by the structural and functional characteristics of muscles [2]. They have been widely used in various research, industrial, and clinical settings [3,4]. Potential applications for signal classification and surface EMG include control of robotic arms and fingers, electric wheelchairs, multifunction prostheses and in particular neural prostheses, virtual keyboard and mouse, navigation in virtual worlds, and more [3].

Among the above basic human-control tasks, reaching and grasping are ubiquitous in everyday life and also serve as human interfaces for controlling robotic systems [5–7]. Identification of hand movements from EMG measurements has been used in video games, robotic exoskeleton devices, power prostheses and more [8–11]. A large number of these studies focus on feature selection for EMG movement classification and include a dimensionality-reduction step followed by machine-learning-based classification.

These studies have suggested that successful classification and pattern recognition of EMG signals require three main steps in the following order: (i) data preprocessing, (ii) feature extraction, and (iii) classification. Common EMG data preprcoessing steps include low- and high-pass filtering, whereas feature extraction is a method of finding intrinsic and meaningful information that may be latent in the EMG signal [12,13]. Over the past few decades, various manual EMG feature-extraction methods have explored in the time and/or frequency domains [14]. Finding optimal feature vectors therefore plays an important role in EMG classification because appropriate feature extraction tends to result in considerably high classification accuracy [12,15].

A common method to extract features from signals is dimensionality reduction, or learning low-dimensional embeddings from samples in high dimensional space [16–18]. Most dimensionality-reduction techniques are linear and relate to Principal Component Analysis (PCA) [19] or Multi-Dimensional Scaling (MDS) [20]. While applying PCA may result in a lower-dimensional representation that captures more relevant information, such linear techniques have various limitations when applied to EMG. They are often less reliable and more sensitive to the number of samples in the training set. In addition, linear techniques by nature model linear relations, which may not describe EMG signals well. Last, linear techniques are global by nature, which means that they cannot preserve local structures in the original feature space [21].

More modern, non-linear dimensionality-reduction techniques include Locally Linear Embedding (LLE) [22]. The LLE algorithm computes the basis of a low-dimensional space, though the dimensionality of the embedding often needs be given as a parameter [23]. Moreover, the output is an embedding for the specific given dataset and not a general mapping from the original to the lower-dimensional space. LLE is also not isometric and often fails by mapping distant points close to each other. Another non-linear technique, ISOMAP, is an extension of MDS that uses geodesic instead of Euclidean distances and can therefore be applied to non-linear manifolds [24]. The geodesic distances between points are approximated by graph distances. Then, MDS is applied on the geodesic distances to compute an embedding that strives to preserve distance between points.

Here we used the Laplacian Eigenmaps algorithm [25]. It computes the normalized graph Laplacian of the adjacency graph of the input data, which is an approximation of the Laplace-Beltrami operator on the manifold. It exploits locality-preserving properties that were first observed in clustering. The Laplacian Eigenmaps algorithm can be viewed as a generalization of LLE, as the two are identical when the weights of the graph are chosen according to the criteria of the latter. Much like LLE, the dimensionality of the manifold also needs to be provided; the computed embeddings are not isometric, and a general mapping between the two spaces is not output. In the past, EMG-based classification using non-linear dimensionality reduction techniques was more often applied to human gait [26,27] than to the more complex reach-and-grasp movements, which also utilize more degrees of freedom [21].

In this study, we used the WAY_EEG_GAL open public dataset, which is freely available (see Materials and Methods) and commonly used to test techniques for decoding during a reach-grasp-lift task. In particular, our aim was to decode the weight of an object (165, 330, or 660 g) from the time-domain EMG data of twelve subjects, who reached and lifed the object. After preprocessing, we automatically extracted the features, reduced the dimensionality, and fed the resulting data into a machine-learning classifier. A key objective of our study was to compare different—linear and nonlinear—dimensionality reduction techniques and different classification techniques over the EMG data. In addition, previous work on the WAY_EEG_GAL dataset included either EEG alone or EEG together with EMG, whereas we wanted to investigate to what extent we could classify the weights in this reach-grasp-lift task using EMG alone. However, we did not focus our study on deep-learning (DL) techniques. Although DL does enable automatic end-to-end learning of preprocessing, feature extraction, and classification modules, DL models are also typically complex—i.e., have many free parameters (or degrees of freedom) to fit—and therefore require large amounts of data to overcome the risk of overfitting those models to specific quirks of the training set. Therfore, they limit the generalizability of the model to an independent test set (although data augmentation might emiliorate these issues) [28].

By directly manipulating EMG signals, our study therefore shifts the focus from manual (human-based) feature engineering to completely automated feature-learning even when only few training samples are available [29–31].

## Materials and method

**Fig 1:**
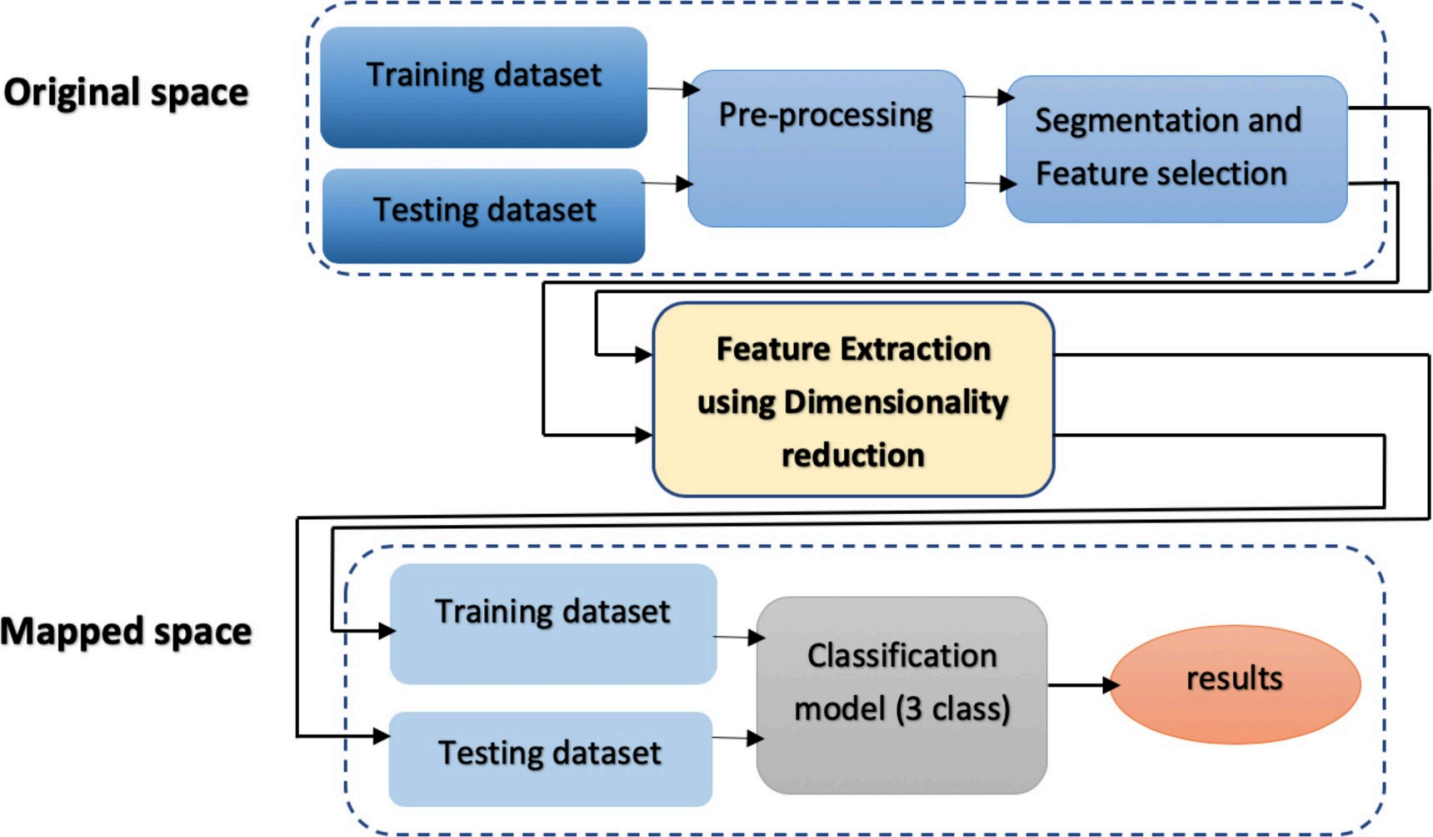
Processing pipeline for EMG signal classification.

**Fig 2:**
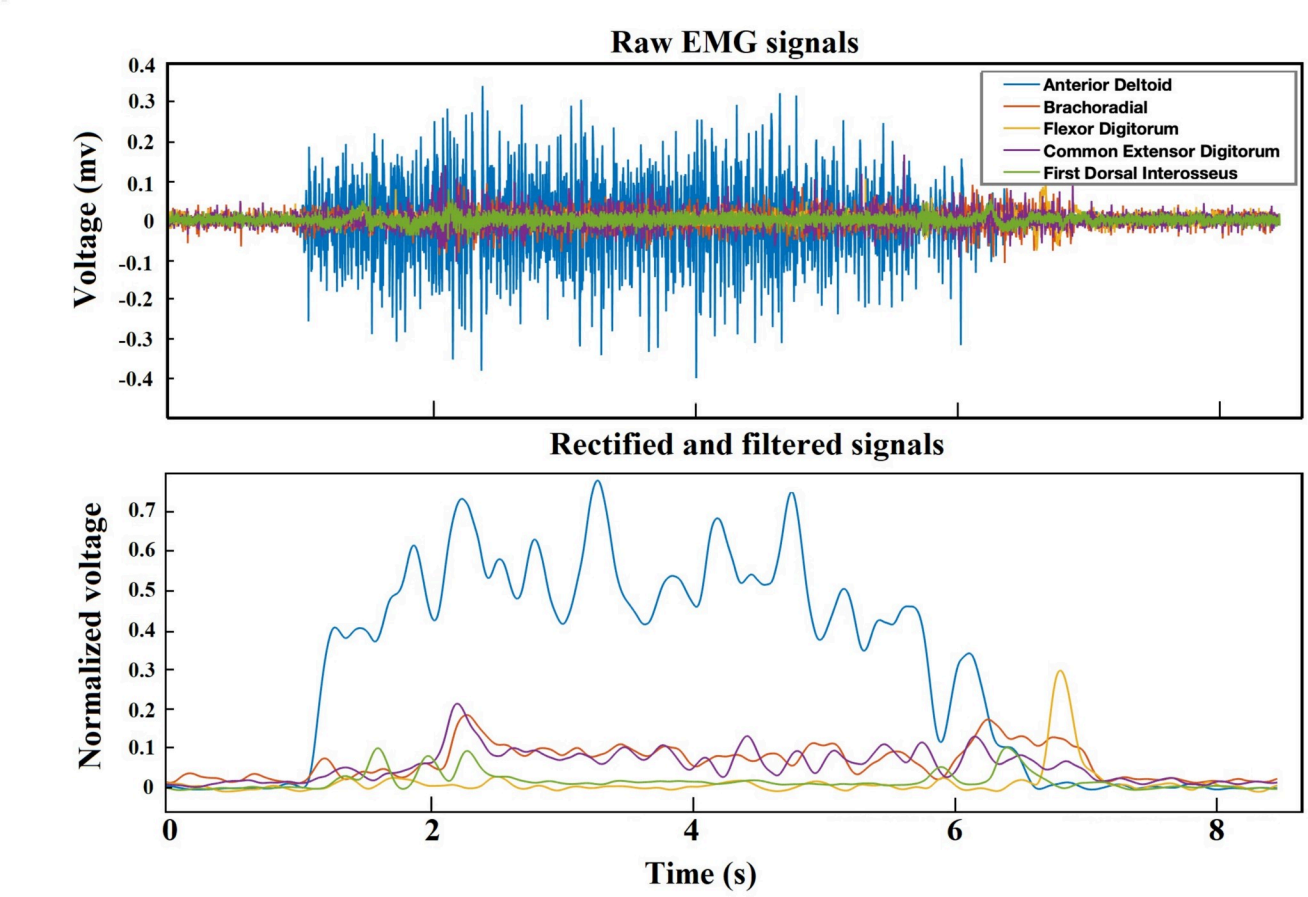
EMG preprocessing. (Top) Pre-processed EMG signals of 5 muscles (Anterior Deltoid, Brachoradial, Flexor Digitorum, Common Extensor Digitorum, and First Dorsal Interosseus). (Bottom) Rectified and filtered EMG signals by band-pass Butterworth filter (4th order) in the 5–450 Hz range on the full-wave rectified and normalized signals from each muscle.

Our methodology for EMG signal classification is illustrated in Fig 1 and detailed below. Briefly, EMG signals were first preprocessed and segmented into the first 8 s of each trial before feature extraction. This segmentation ensured that the subject started from home position and returned to the home position, removing noise after returning to the home position (Fig 2). The components corresponding to the highest eigenvalues from the output of the dimensionality-reduction algorithms were extracted as the dominant features. Thereafter, these intrinsic features were used for classification.

### Dataset

**Fig 3:**
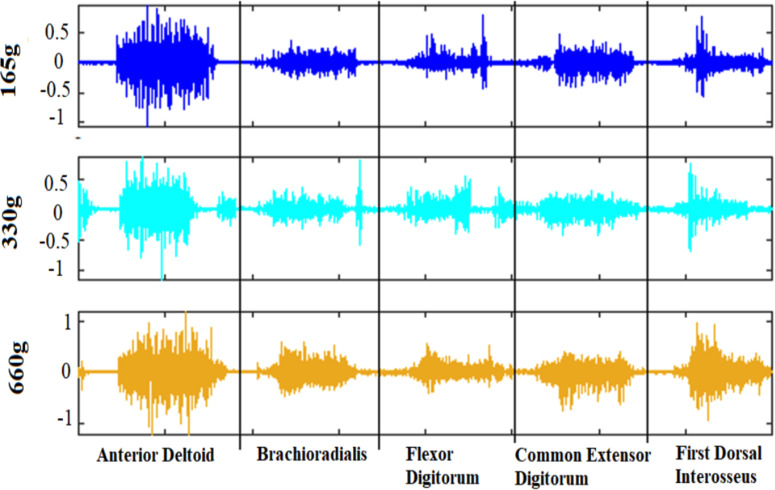
Raw EMG signals of 5 muscles. (Anterior Deltoid, Brachioradialis, Flexor Digitorum, Common Extensor Digitorum, and First Dorsal Interosseous) for 3 different weights (165, 330, and 660 g).

The WAY_EEG_GAL dataset is freely available and has become somewhat of a benchmark to test techniques that decode sensation, intention, and action from surface EMG and scalp EEG in humans performing a reach-grasp-lift task (https://doi.org/10.6084/m9.figshare.c.988376) [32]. Here we focus exclusively on EMG data. The EMG signals were sampled at 4 kHz. In each trial, the participants rested their hand in the home position. Then they were cued to reach for the object, grasp it with the thumb and index finger, lift it straight up in the air and hold it for a few of seconds. They were then instructed to put the object back on the support surface, let go of it, and return the hand to a designated home position [32]. The state of the LED indicated to the participant to start and terminate a trial. The object’s weight varied between 165, 330, and 660 g and the surface material varied between sandpaper, suede, or silk. We used all available 2,645 trials of EMG signals, across all 12 subjects, including trials with different weights (840 trials for 165 g, 1122 trials for 330 g, and 683 trials for 660 g). The number of trials for each subject was 220 or 221, and the highest imbalance-ratio between classes for any subject was 0.61 (Table 1). The material in all trials was always sandpaper, as per the original design of the experiment [32]. Five EMG electrodes recorded the activity from 5 muscles (Figs 2 and 3).

**Table 1:** The number of trials for each class and each subject.

ID	165g	330g	660g	imbalance-ratio	Total trials for each subject
**Subject 1**	70	93	57	0.61	220
**Subject 2**	70	94	57	0.61	221
**Subject 3**	70	93	57	0.61	220
**Subject 4**	70	94	57	0.61	221
**Subject 5**	70	94	57	0.61	221
**Subject 6**	70	93	56	0.60	219
**Subject 7**	70	94	57	0.61	221
**Subject 8**	70	93	57	0.61	220
**Subject 9**	70	93	57	0.61	220
**Subject 10**	70	94	57	0.61	221
**Subject 11**	70	94	57	0.61	221
**Subject 12**	70	93	57	0.61	220
**Total**	840	1122	683	**Mean: 0.61**	**2645**

### Preprocessing

All processing was carried out on a PC (3.4 GHz Intel® CoreTM i7-6700 CPU) using Python 3 and MATLAB 2019b.

EMG signals are typically contaminated by various types of noise and artifacts. Therefore, preprocessing prior to feature extraction was important. We used a band-pass Butterworth filter (4th order) in the 5–450 Hz range on the full-wave rectified and normalized signals from each muscle (Fig 2).

### Segmentation and feature selection

The time required to reach, grasp, and lift varied among trials and subjects. So, we focused on the first 8 seconds for every trial. Doing so also removed noise that appeared at the end of the trial, after the subject returned their hand to the home position. For feature selection, we concatenated the signals of the 5 muscles (as in Fig 3). We then subsampled, taking every 5th sample for increased processing speed (lowpass filtering was already carried out before the subsampling, as part of the band-pass filter during preprocessing). We ended up with 5 x 8 x 800 (muscle x time (second) x samples) = 32,000 features.

### Feature extraction using dimensionality reduction

EMG signals are complex, high-dimensional, and non-linear and hence hard to study in their original form. Effort has therefore been put into finding meaningful, low-dimensional features of these signals. Classical dimensionality-reduction techniques include linear methods, such as principal component analysis (PCA) [33] and linear discriminant analysis (LDA) [34]. These techniques preserve global structure of the data but at the cost of obscuring local features and preventing any local manipulation of the data.

In contrast, manifold learning is a non-linear technique for recovering a low-dimensional representation from high-dimensional data [23,35]. The literature on manifold learning is dominated by spectral methods. These have a characteristic computational pattern. The first step involves the computation of the k-nearest neighbors (k-NN) of all N data points. Then, an N×N square matrix is populated using some geometric principle. This characterizes the nature of the desired low-dimensional embedding. The eigenvalue decomposition of this matrix is then used to obtain the low-dimensional representation of the manifold.

A trade-off between preserving local and global structures must often be made when inferring the low-dimensional representation. Manifold learning techniques such as Locally Linear Embedding (LLE) [22], Laplacian Eigenmaps [25], and t-Distributed Stochastic Neighbor Embedding (t-SNE) [36] are considered to be local methods because they are designed to minimize some form of local distortion and hence result in an embedding that preserves locality. Methods such as ISOMAP [23] are considered global because they preserve all geodesic distances in the low-dimensional embedding. All spectral techniques are parameterless (except for neighborhood size; see below) and hence do not characterize the map that generates them. In this study, we compared different algorithms for manifold learning—Global: ISOMAP; and local: LLE, t-SNE, Laplacian Eigenmaps—and further compared them with linear dimensionality-reduction techniques, PCA and LDA (the latter is the only supervised dimensionality-reduction technique). In the next section, we explain the Laplacian Eigenmaps algorithm in more details.

#### The Laplacian Eigenmap algorithm

The Laplacian Eigenmap algorithm plays a larger role in this study, hence we describe it in more detail, following Belkin et al. (see [25]). Given *k* points *x*_1_,…,*x*_*k*_ in *R*^*l*^, it finds a set of points *y*_1_,…,*y*_*k*_ in ℝ^*m*^ (*m*≪*l*) such that *y*_*i*_ represents *x*_*i*_. Therefore, *x*_1_,…,*x*_*k*_∈*M* and *M* is a manifold embedded in ℝ^*l*^. The Laplacian Eigenmaps (spectral embedding) is based on the following steps:

Algorithm 1. Laplacian Eigenmaps

Input: High-dimensional data-points of the manifold:

{*x*_*i*_∈ℝ^*l*^}, *i* = 1,2,…,*k*

Output: Low-dimensional embeddings of data points:

{*y*_*i*_∈ℝ^*m*^}, *m*≪*l*, *i* = 1,2,…,*k*

Step1. Constructing the graph:

We put an edge between nodes *i* and *j* if *x*_*i*_ and *x*_*j*_ are *n*-nearest neighbors. Thus, nodes *i* and *j* are connected by an edge if *i* is among the *n*-nearest neighbors of *j*, or *j* is among *n*-nearest neighbors of *i*. This then leaves us with a connected graph.

Step 2. Choosing the weights. There are two possible ways for choosing the weights:

a. Heat Kernel: $W_{ji}=e^{-\frac{{\left\|x_{i}-x_{j}\right\|}^{2}}{2\sigma^{2}}}$, if vertices *i* and *j* are connected by an edge; and *W*_*ji*_ = 0, if vertices *i* and *j* are not connected by an edge. The only parameter in the Heat-Kernel equation is *σ*, which defines the extent to which distant neighbors influence the embedding of each point. The choice of parameter *σ* is data-dependent and is typically tuned empirically.

b. Simple-Minded: *W*_*ij*_ = 1,if vertices *i* and *j* are connected by an edge and *W*_*ji*_ = 0 if vertices *i* and *j* are not connected by an edge.

Step 3. Eigenmaps: Compute eigenvalues (*λ*) and eigenvectors (f) for the generalized eigenvector problem:

*Lf* = *λDf*,

where D is a diagonal weight matrix, and its elements are column (or row, since W is symmetric) sums of *W*.

*D*_*ii*_ = ∑_*j*_*W*_*ji*_, *L* = *D*–*W* is the Laplacian matrix (symmetric, positive semidefinite).

We leave out the eigenvector corresponding to eigenvalue 0 and use the next *m* eigenvectors for embedding in *m*-dimensional Euclidean space: *x*_*i*_→*f*_1_(*i*),…,*f*_*m*_(*i*). The *m* eigenvectors will be considered features of the dataset.

**Fig 4:**
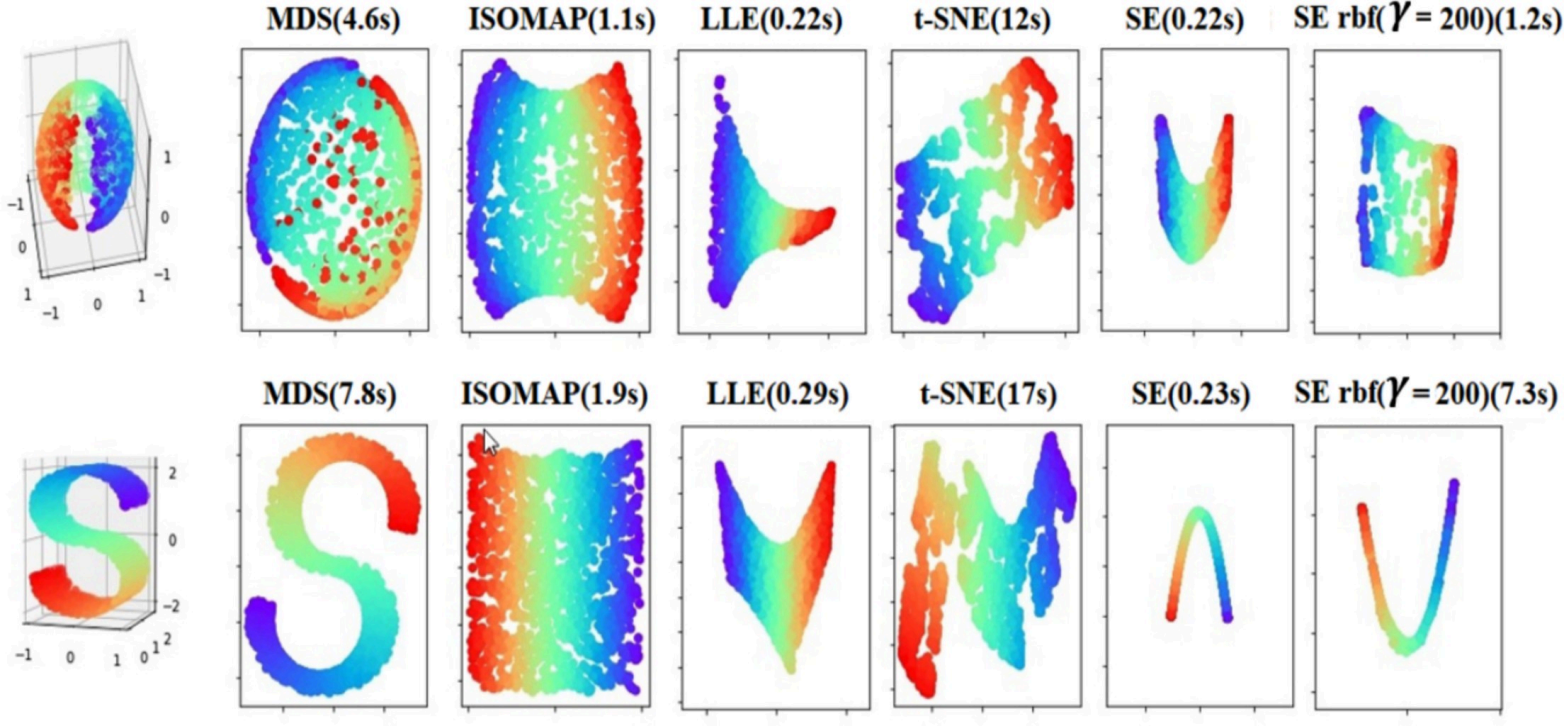
Manifold learning techniques. MDS, ISOMAP, LLE, t-SNE, and Spectral embedding (SE) or Laplacian Eigenmaps on 2000 points randomly distributed on the surface of a sphere. Computation time in seconds is given after each method’s name in parentheses. The first column for SE is simple-minded constructing weight matrix and the last column is for heat kernel.

The core algorithm is relatively simple. It has a few local computations (in the matrix) and one solution to the sparse eigenvalue problem. The solution reflects the intrinsic geometric structure of the manifold. It requires a search for neighboring points in a high-dimensional space. The justification for the algorithm comes from the role of the Laplace Beltrami operator in providing an optimal embedding for the manifold. The manifold is approximated by the adjacency graph computed from the data points. The Laplace Beltrami operator is approximated by the weighted Laplacian of the adjacency graph, with weights chosen appropriately. The key role of the Laplace Beltrami operator in the heat equation enables us to use the heat kernel to choose the weight decay function in a principled manner. Thus, the embedding maps for the data approximate the eigenmaps of the Laplace Beltrami operator, which are maps intrinsically defined on the entire manifold. For more information about the justification for Laplacian algorithm and the role of the Laplace Beltrami operator in providing an optimal embedding, see Supplementary Methods. The low dimensional representation of the data set that optimally preserves local neighborhood-information may be viewed as a discrete approximation to a continuous map that naturally arises from the geometry of the manifold. It is worth highlighting some aspects of Laplacian Eigenmaps here: 1) The algorithm reflects the intrinsic geometric structure of the manifold, which is simple with few local computations and one sparse eigenvalue problem. 2) The justification for the algorithm comes from the role of the Laplace-Beltrami operator in providing an optimal embedding for the manifold. The key role of the Laplace-Beltrami operator in the heat equation that enables us to use the heat kernel is to choose the weight decay function in a principled manner. Thus, the embedding maps for the data approximate the Eigenmaps of the Laplace-Beltrami operator, which are maps that intrinsically depend on the entire manifold. 3) The locality preserving character of the Laplacian Eigenmap algorithm makes it relatively insensitive to outliers and noise. Close connections to spectral clustering algorithms were developed in machine learning and computer vision. To help gain intuition about manifold-learning algorithms, we demonstrate their use on a simple, spherical dataset (2000 random points on the surface of a 3D sphere) and on a “Swiss roll” (The 2000 points chosen at random from the Swiss roll; Fig 4). We used the Scikit-learn Python package [37] and Matlab toolbox [38,39] for dimensionality reduction. Laplacian Eigenmaps are termed Spectral Embedding (SE) in Scikit-learn and the embedding is not strictly the adjacency matrix of a graph but more generally an affinity or similarity matrix between samples [40]. It has 2 different methods (heat kernel and simple-minded) for constructing the weight matrix. The kernel function for the Heat-Kernel ($W_{ij}=e^{-\frac{{\left\|x_{i}-x_{j}\right\|}^{2}}{2\sigma^{2}}}$) in this package is a Gaussian radial basis function kernel (RBF) with $\gamma =\frac{1}{2\sigma^{2}}$, where γ is a parameter that sets the “spread” of the kernel. The results of various manifold-learning techniques for 8 neighbors in 2D space are shown in Fig 4. Laplacian Eigenmaps (simple-minded), or SE, is the fastest algorithm; the computation time for SE-rbf is 5.5 times longer for a sphere and 31.7 times longer for a Swiss roll. It appears that the construction of the weight matrix drives this difference in computation time. For more information about the properties of techniques for dimensionality reduction, see Supplementary Methods.

**Fig 5:**
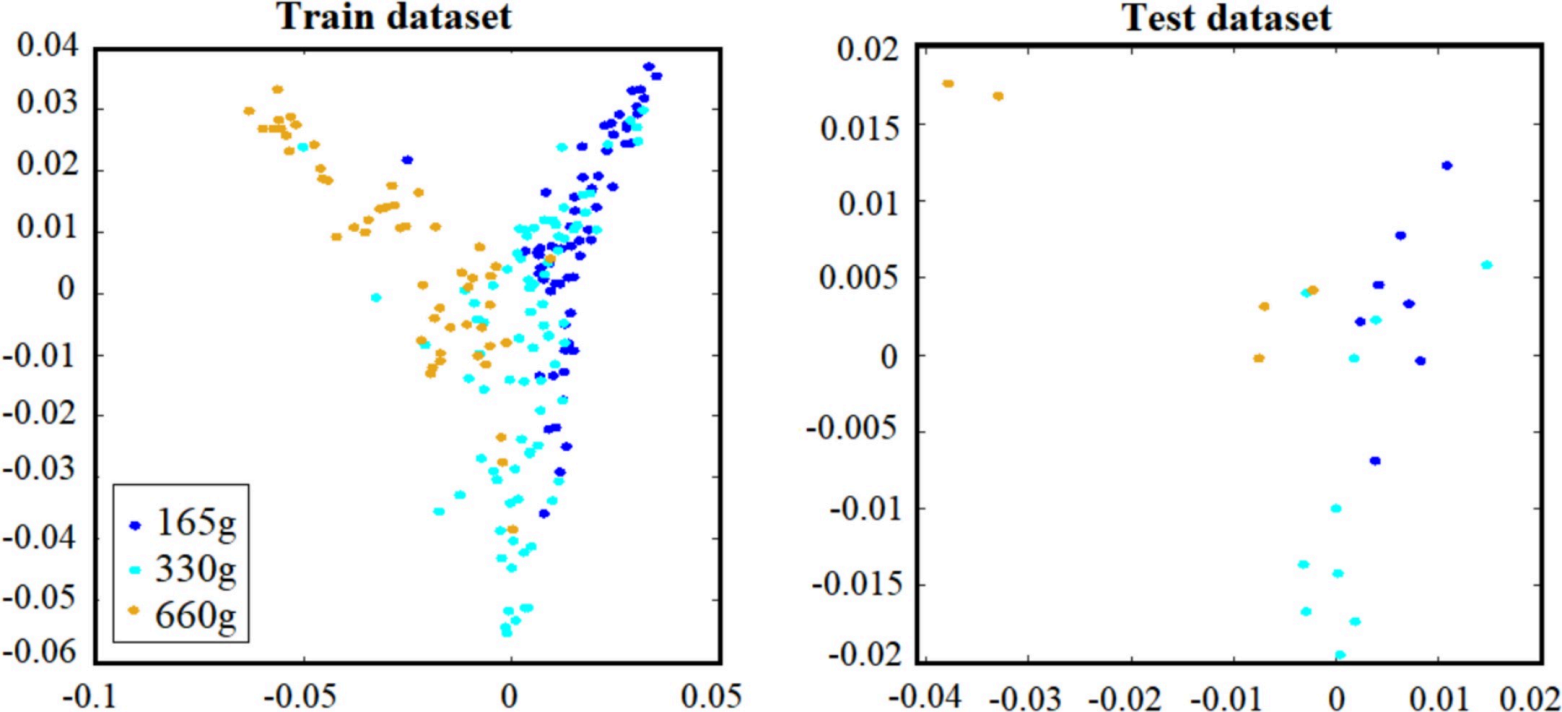
Example of an out-of-sample extension via the Nyström approximation in embedded space by Laplacian Eigenmaps with k = 8 and σ = 1. The train/test ratio was 90%/10%.

An important requirement for dimensionality reduction techniques is the ability to embed new high-dimensional datapoints into an existing low-dimensional data representation. However, there is no explicit projection function between the original data and their low dimensional representations in the original LE algorithm, which makes out-of-sample extension difficult. To find projection of any additional samples, LE needs to be run on all the data together with the additional samples, resulting in considerable computational cost, especially when applying it to large scale data pattern recognition. Fortunately, various methods have been developed to mitigate the out-of-sample problem [5]. Nyström approximation supports out-of-sample extensions for spectral techniques such as ISOMAP, LLE, and Laplacian Eigenmaps. In Supplementary Methods, we explain Nyström approximation in greater details. In Fig 5 we depict the embedding of additional, out-of-sample points (there termed “test dataset”). As is apparent, the out-of-sample points are mapped to plausible locations in the low-dimensional space. For more information about the out-of-sample extension, see Supplementary Methods.

### Classification

After finding an optimal feature set, we tested commonly used classification algorithms: k-NN [41], linear and RBF SVM [30] (C = 32, *γ* = 0.01 for RBF SVM), and Random Forest [42]. We evaluated the performance of each classifier on the data after running the above dimensionality-reduction techniques. We ran the analysis on each subject separately. The dataset was divided into disjoint training and testing sets, which consisted of 90% and 10% of the total trials, respectively. For each subject, we further ran 10-fold cross-validation on the training dataset. Table 1 shows the details of trials for each class and subject. The results we report are therefore averaged over all subjects on the testing dataset.

Another common method for EMG classification, on top of those above, is deep learning [43]. We tested several deep learning architectures on our selected feature set. However, we ran into severe overfitting issues resulting in accuracies very close to chance level. This result is likely due to the relatively small features-to-samples ratio for our dataset. Consequently, we did not include deep-learning results in our analyses.

As the dataset was imbalanced (the highest imbalanced ratio was 0.6), we used the F1-score as a metric of accuracy [44]. The F1-score provides a way to combine both precision and recall into a single measure that captures both properties. Once precision and recall have been calculated for a binary or multiclass classification problem, the two scores can be combined into the calculation of the F1-score.

$$F1‐score=\frac{2∙Precision∙Recall}{Precision+Recall},$$

where $Precision=\frac{TP}{\left(TP+FP\right)}$ and $Recall=\frac{TP}{\left(TP+FN\right)}$. Here TP is number of true positives, FP is number of false positives, and FN is number of false negatives. This is the harmonic mean of the two fractions. The F1-Score is a very common metric for imbalanced classification problems [45].

### Running-window analysis

In this section we describe an additional, running-window analysis that we performed on this dataset. This section helps to get more insight into the temporal dynamics of the current model’s classification accuracy. In the analysis, we used a 100 ms sliding window with a step size of 40 ms. We tested various step sizes (between 10 and 50 ms) and 40 ms resulted in the best visualization (though the visual differences between the step sizes were minute).

We applied the proposed pipeline in a sliding-window manner to estimate the extent to which the prediction accuracy would be stable over consecutive time windows. Laplacian Eigenmaps (simple-minded k = 8), 120 dimensions after embedding with k-NN (number of neighbors k = 8), was applied on the preprocessed EMG signal. The minimum length of the sliding window that is possible to run on this dataset is 100 ms. Below this length, the adjacency graph of the input data appears not to be fully connected (Supplementary Methods). For example, a row (and a column, since this is symmetric) can be all zeros and therefore one of the nodes will not be connected, resulting in a warning.

We therefore segmented the dataset based on the events (onset of touching the object, LED on, LED off). There was variability among subjects’ speed in this task. The minimum time across subjects was 0.74 s before touching the object and 1.82 s after touching the object.

## Results

### Parameter settings

**Fig 6:**
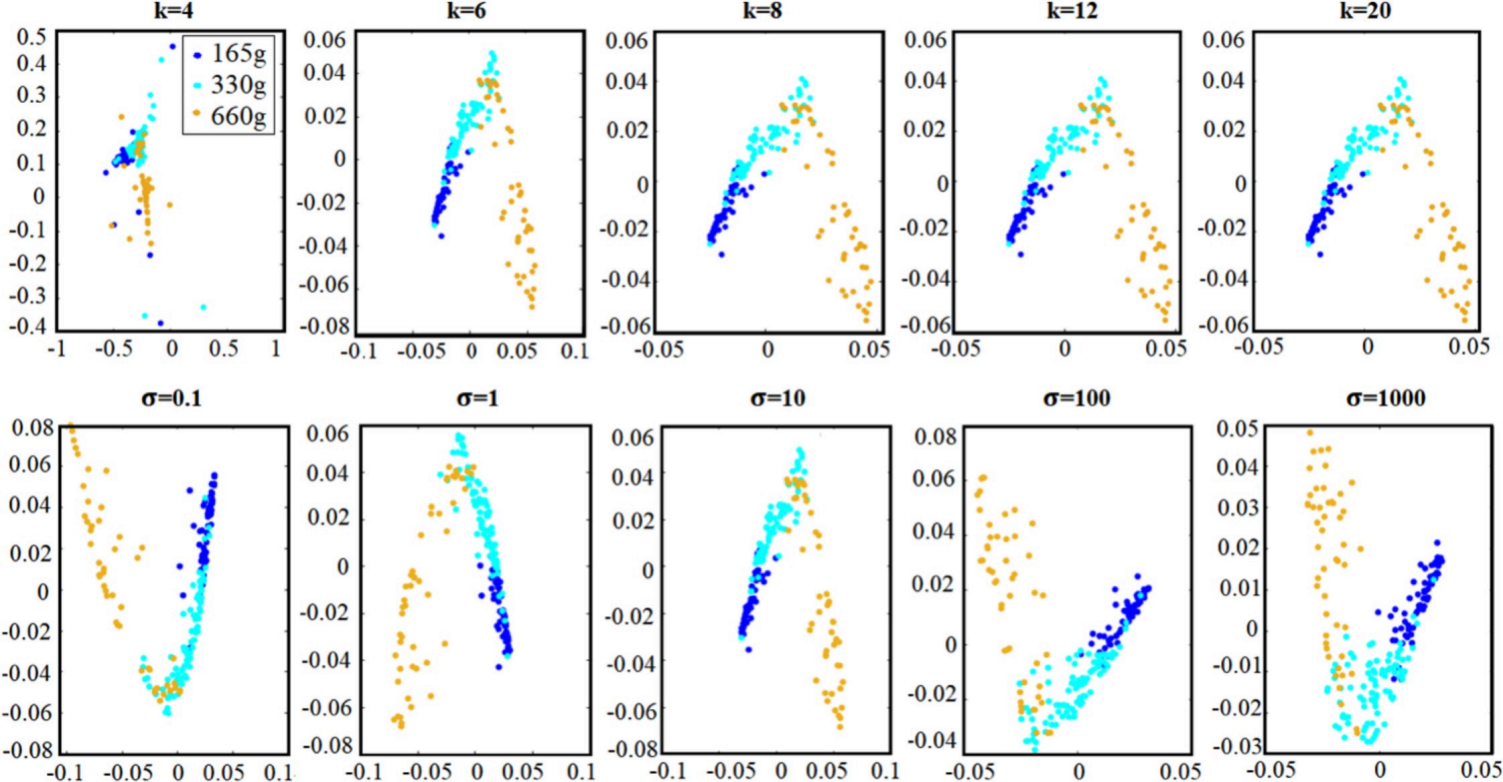
Visualization of the effect of k (top row) and *σ* (bottom row) on the training dataset for Subject 1 in the embedded space.

The proposed framework has 2 parameters: the number of nearest neighbors in Laplacian Eigenmaps to construct the Laplacian matrix (either using the direct number of neighbors, *k*, or using a heat kernel approach, ***σ***) and the number of eigenvectors used for data mapping, i.e. the dimensions of the mapped space. The number of nearest neighbors in Laplacian eigenmaps, *k*, was tuned to 4, 5, 6, …, 20—i.e., using a grid search. We also tested values of ***σ*** in the range 0.1, 1, 10, 100,1000, again using a grid search. Fig 6 shows the effect of different *k* and ***σ*** values on the training dataset for Subject1. The number of eigenvectors or dimensions is tuned in the range of 1,5, 10, …, length (training trial), once more using a grid search. (see S1 Table in S1 Text. Properties of techniques for dimensionality reduction)

**Fig 7:**
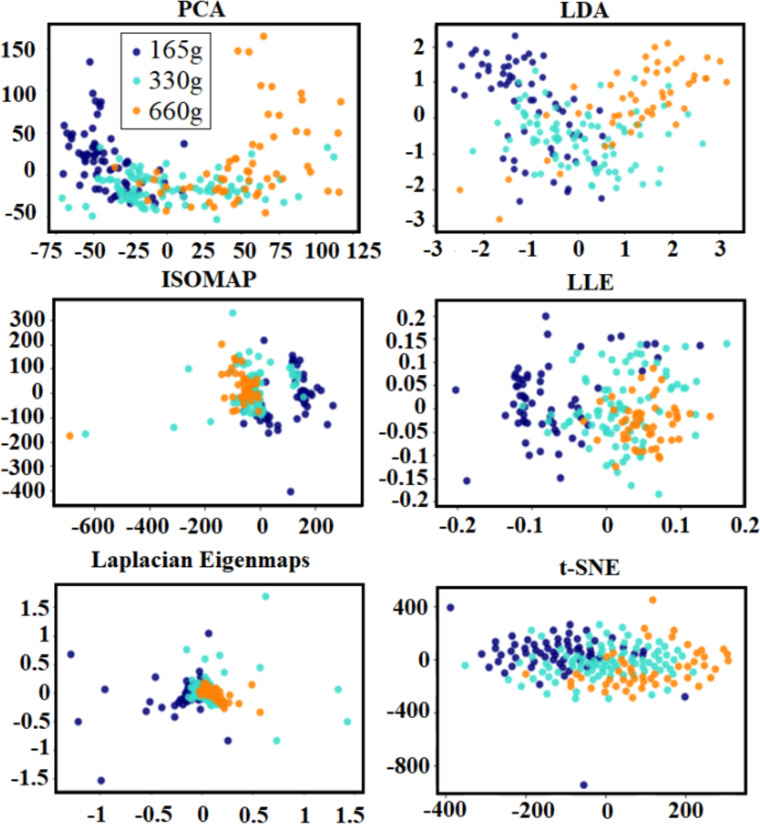
Visualization of the embedding process. EMG visualization using the 2 most prominent components of different dimensionality-reduction technique (The x-axis is the most prominent component, and the y-axis is the second most prominent component).

Table 2 shows the optimal number of eigenvectors for each dimensionality-reduction method across all subjects. As a sanity check, we also used the maximum likelihood estimator (MLE) as the intrinsic dimensionality estimator in the Matlab toolbox for dimensionality reduction [39]. The number of eigenvectors varied between 110 and 170 over the 12 subjects for different dimensionality-reduction techniques. We also visualized the embedded EMG using our six dimensionality-redution methods (PCA, LDA, ISOMAP, LLE, Laplacian Eigenmaps, and t-SNE). Fig 7 shows the 2 most prominent components for each of these methods.

**Table 2:** The number of dimensions that leads to the highest F1-score (obtained using grid search; for k-NN, which was the best classifier; see also Fig 8) vs. different dimensionality reduction techniques for each subject. The mean and standard error (SE) over all subjects for the different methods are also given.

ID	PCA	ISOMAP	LLE	LE(simple minded)	LE(rbf)	t-SNE
**Subject 1**	160	180	135	115	120	180
**Subject 2**	170	155	110	115	110	155
**Subject 3**	165	175	125	125	125	170
**Subject 4**	190	180	90	115	90	190
**Subject 5**	170	180	120	120	105	175
**Subject 6**	155	130	110	115	145	130
**Subject 7**	175	120	100	140	120	175
**Subject 8**	125	145	120	120	125	185
**Subject 9**	110	100	95	125	95	140
**Subject 10**	170	160	135	140	155	185
**Subject 11**	170	185	110	125	105	170
**Subject 12**	165	190	95	115	110	140
**[Min Max]**	**[110 190]**	**[100 190]**	**[90 135]**	**[115 140]**	**[90 155]**	**[130 190]**
**Mean± SE**	**160.4±6.4**	**158.3±8.4**	**112.1±4.4**	**122.5±2.6**	**117.9±5.5**	**166.3±5.8**

### Classifier performance

**Fig 8:**
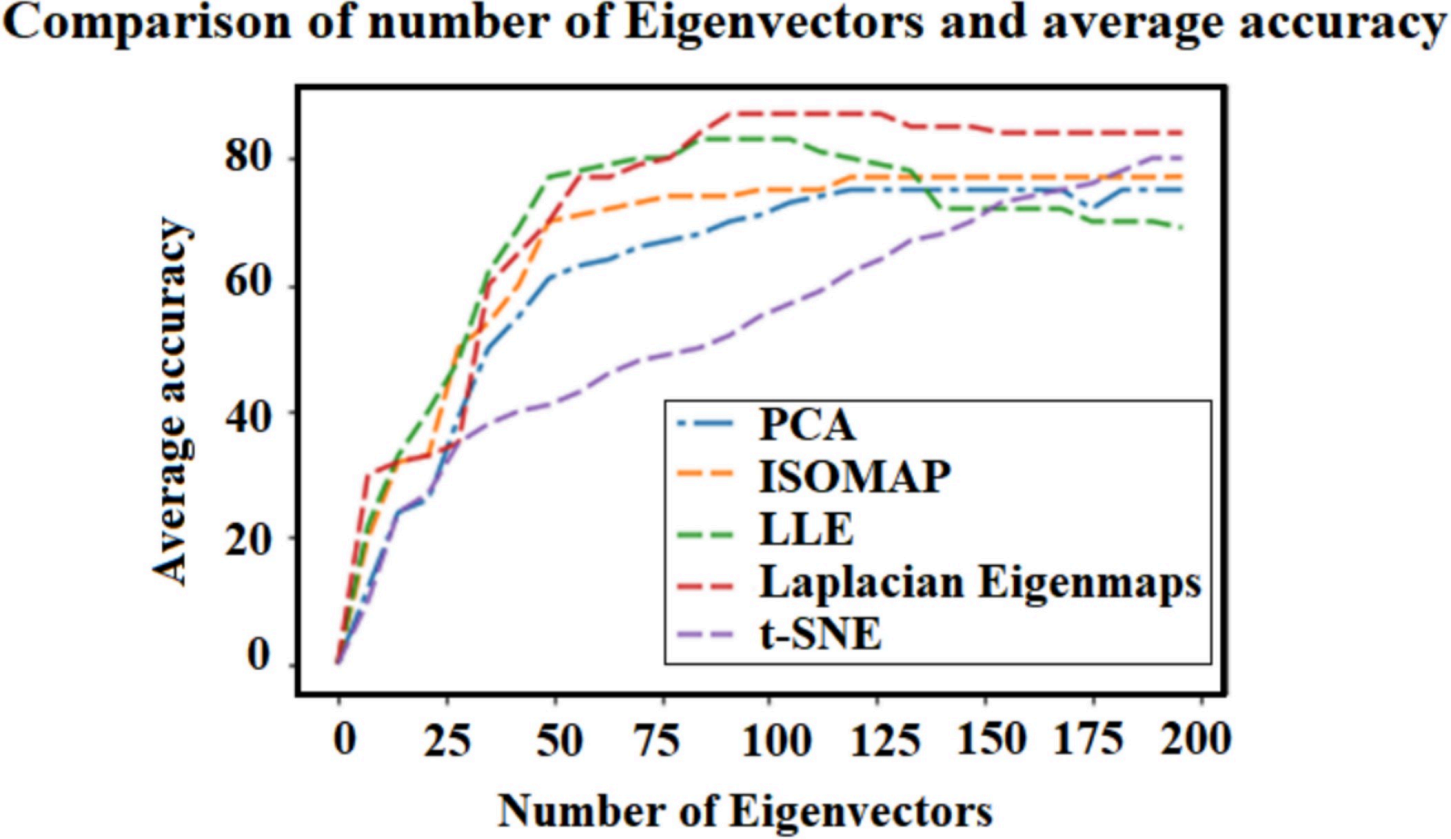
Average accuracy (across all 12 subject) as a function of the number of eigenvectors obtained with different dimensionality reduction techniques for the best classifier—k-NN (see also Table 3). We included PCA, ISOMAP, LLE, Laplacian Eigenmaps, and t-SNE. The LDA dimensionality-reduction technique was not included in these curves because the maximal dimension for LDA is equal to the number of classes minus one.

We computed the average accuracy using PCA, ISOMAP, LLE, Laplacian Eigenmaps, and t-SNE for the classifier that produced the highest accuracy—k-NN (Fig 8). In Table 3, the performance of Laplacian Eigenmaps (simple-minded) and different classifiers vs. different number of neighbors (k) is shown. It demonstrates that k = 8 fits well for this dataset.

**Table 3:** Performance of Laplacian Eigenmaps (simple-minded) and different classifiers vs. different number of neighbors (k) on the EMG signals (F1 score ± SE).

Different number of neighbors(k)	4	6	8	10	12	15	20
**Laplacian Eigenmaps (simple-minded) + k-NN**	43.42(±9.2)%	71.98(±4.9)%	88.2(±3.5)%	79.32(±6.7)%	71.76(±4.5)%	63.64(±7.8)%	58.31(±6.3)%
**Laplacian Eigenmaps (simple-minded) + RBF SVM**	32.88(±6.4)%	74.68(±2.7)%	77.6(±2.3)%	76.98(±3.1)%	76.46(±8.5)%	73.52(±4.3)%	68.23(±8.1)%
**Laplacian Eigenmaps (simple-minded) + Linear SVM**	43.42(±5.3)%	62.98(±3.9)%	63.6(±2.2)%	63.59(±2.7)%	63.06(±1.5)%	63.64(±7.8)%	58.31(±6.2)%
**Laplacian Eigenmaps (simple-minded) + Random Forest**	59.21(±3.2)%	72.28(±4.3)%	79.5(±2.8)%	78.54(±2.4)%	77.76(±4.5)%	77.64(±3.9)%	68.22(±2.3)%

Table 4 details the prediction accuracies of the different classification algorithms on the test set for the various dimensionality-reduction techniques over all 12 subjects. On average, Laplacian Eigenmaps (especially with a heat kernel) is the algorithm with the highest accuracy across all dimensionality-reduction methods—78.15%. And k-NN is the classification method resulting in the highest mean accuracy across all classification algorithms—80% on average—and significantly higher than linear SVM and RBF-SVM and marginally higher than Random Forest (repeated-measures ANOVA F(3) = 15.5, p<0.001; post-hoc t-tests suggest all comparisons are significant at the 0.05 level except Random Forest vs. k-NN, which was p = 0.053; and RBF-SVM vs. Random Forest, p = 0.685—see S4 and S5 Tables in **S1 Text**; we further found no evidence that the pairwise differences are not normally distributed—Shapiro-Wilk test was not significant). Interestingly, the intersection of Laplacian Eigenmaps and k-NN has the highest overall accuracy, at 88%. We also ran a statistical analysis on the different dimension reduction techniques to compare the linear and non-linear techniques. However, there wasn’t significant difference between them, maybe because of the low number of subjects (see S2 and S3 Tables in **S1 Text**).

**Table 4:** Performance of different classifiers vs. different dimensionality-reduction methods on EMG signals (F1 score ±SE). See S4 Table in S1 Text for post-hoc t-tests for this table.

	k-NN	RBF SVM	Linear SVM	Random Forest	Average (%)
**PCA**	75.3(±2.8)%	64.3(±1.2)%	63.5(±4.9)%	75.4(±3.2)%	**69.62(±3.3)%**
**LDA**	78.2(±15.3)%	72.7(±13.2)%	67.2(±12.2)%	76.2(±9.3)%	**73.57(±2.4)%**
**ISOMAP**	77.4(±7.2)%	74.4(±2.2)%	57.7(±3.9)%	73.9(±4.9)%	**70.85(±4.4)%**
**LLE**	84.6(±7.9)%	82.3(±4.1)%	58.5(±4.1)%	76.7(±3.8)%	**75.52(±5.9)%**
**Laplacian Eigenmaps (simple-minded k = 8)**	88.2(±3.5)%	78.2(±2.3)%	63.6(±2.2)%	72.6(±2.8)%	**77.7(±3.8)%**
**Laplacian Eigenmaps (rbf, σ = 10)**	84.2(±3.9)%	71.2(±5.3)%	61.3(±4.7)%	79.9(±2.9)%	**78.15(±3.7)%**
**t-SNE**	75.8(±4.2)%	73.2(±2.1)%	71.1(±8.3)%	69.3(±8.7)%	**72.35(±1.3)%**
**Average (±*SE*))%**	**80.53(±1.9)%**	**75.24(±2.3)%**	**65.24(±2.1)%**	**74.85(±1.2)%**	

### Evolution of classification accuracy over time

**Fig 9:**
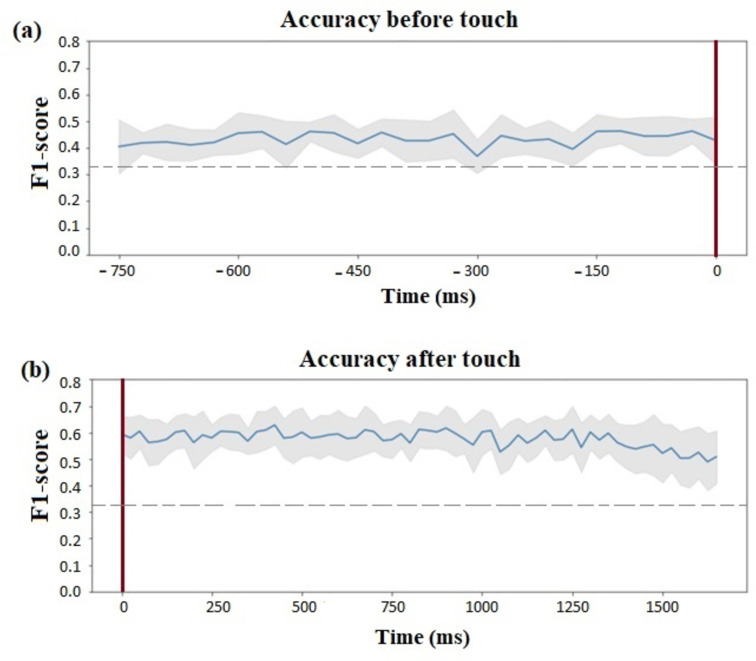
Sliding-window analysis of Laplacian Eigenmap dimensionality reduction and k-NN classification before touching the object (a) and after touching it (b). We used a 100 ms sliding window with a step size of 40 ms. In both panels, the onset of touching the object is designated by a vertical red line at time 0.

So far, we focused on optimizing the dimensionality reduction and classification accuracy on the entire movement duration. However, another interesting aspect of this dataset is the evolution of the dimensionality reduction and classification accuracy over time within each trial. A running-window analysis of our best combination of dimensionality reduction technique and classification method (Laplacian Eigenmap and k-NN) suggests that there is little to no information in the EMG of the muscles before the subject touches the object (Fig 9A). The mean accuracy over our 100 ms window during that time was 43.39% (±9.79). It is not surprising that the accuracy is slightly above chance, as some of the experiment was carried out in a blocked design; hence, the weight often did not change between consecutive trials [46]. So, subjects may therefore have begun preparing their hand posture while reaching for the object based on the weight they anticipated from the previous trial. And we were able to capture this preparatory muscle activity with our algorithm.

Perhaps more interesting is the running-window analysis *after* the subjects grasped the object. With such a small window, we expected a much lower accuracy than that over the entire movement window. Indeed, the mean accuracy was only 57.65% (±11.59). But, interestingly, the accuracy was above chance level already in the first 100 ms window (Fig 9B). And it was generally stable throughout much of the duration when the subject held the object; though there appears to have been a small decrease in accuracy toward the end of that time duration.

## Discussion

Our goal in this study was to decode to which of 3 weight classes an object in a reach-grasp-lift task belonged using only EMG data from the arm and hand. In particular, we compared the performances of various linear and non-linear dimensionality-reduction techniques, combined with several classification methods. We worked on pre-processed EMG signals directly, automatically extracting the features for the classification phase. The dimensionality-reduction algorithms we used lowered the dimensionality of our data from 32,000 to less than 200—i.e., more than 160-fold. We then applied various classification techniques on this 3-way classification problem and discovered that the combination of Laplacian Eigenmaps (simple-minded, k = 8) with the k-NN classifier resulted in the highest classification accuracy (F1 score 88.2±3.5%). As a result, we used automatic feature-extraction directly from the pre-processed EMG time-domain signal [20,47–52]. Importantly, our approach to extract features from EMG signal resulted in relatively high decoding accuracy.

Other studies that relied on the same dataset that we used mostly focused on EEG [47–50]. However, Cisotto et al. used both EEG and EMG to classify the same dataset [51]; though they attempted classification of only 2 of the 3 available classes (the most extreme weights: 165 and 660 gr). They also reported their results in terms of accuracy, even though their classes were imbalanced (imbalance ratio of 0.81 between the number of trials in the 2 classes). They reported a maximal accuracy of 94% (using only the Brachoradial muscle). Running our analysis as is (using all muscles and without any parameter optimization) with only the 2 weight classes they used, and a reporting accuracy instead of F1 score, we get an accuracy of 90.9±2.5%. This accuracy is statistically indistinguishable from theirs (t-test: t(11) = -1.33, p = 0.21). Therefore, even though we used only EMG and not EEG, and we did not focus our analysis on a binary classification problem, we were able to achieve comparable results. These might be due to the superiority of our method—perhaps our automatic feature extraction or our dimensionality-reduction algorithm. Another, not mutually exclusive, reason might be that the high classification accuracy that Cisotto et al. we were able to achieve owes much to the EMG signals that they used in conjunction with the EEG signals [51]. Hence, at least for this dataset, the addition of the EEG signals may not have added that much to the decoding accuracy.

The increasing adoption of DL tehcniques in machine learning is shifting the focus from feature engineering to feature learning [8,52]. Nevertheless, the black-box nature of DL makes it hard to understand what information is learned by the network and how it relates to handcrafted features. At the same time, the application of DL on insufficiently large datasets risks overfitting. In additional, the high variability of EMG recordings between participants often makes deep-learned features generalize poorly across subjects.

The range of mean accuracies among the dimensionality reduction algorithms we used was 70–78% (Table 4). Interestingly, Laplacian Eigenmaps not only performed best on average; its simple-minded version also generally required the shortest computing time (see Materials and Methods). The average accuracies of the different classifiers varied from 65.24% to 80.53%. It appears that the linearity of linear-SVM was detrimental for EMG signal decoding, while the most non-linear technique, k-NN, faired best.

It also appears that dimensionality-reduction techniques relying on local embedding were better for this dataset than those that used global embedding. Such local methods strive to map nearby samples on the original manifold to nearby samples in the low-dimensional space (and vice versa for far away samples). Global methods, in contrast, strive for a faithful representation of the data’s global structure. As reach-grasp-lift motion is composed of different phases of movement, local methods may better preserve the varying geometry across phases. Local methods are also computationally more efficient, involving only sparse matrix computations. It may further not be surprising that k-NN works best with local dimensionality-reduction methods. These methods keep nearby samples close to each other, facilitating nearest-neighbor approaches like k-NN.

Our method, therefore, resulted in relatively high accuracy on 3-way classification while maintaining automatic feature extraction. What is more, the methodology proposed in this paper is well suited to real-time operation, potentially in combination with EEG [53], because the computational load in training and testing the model is relatively low. In addition, the variability in many datasets could be due to just a small number of factors. If that is the case, the samples from these datasets may well lie on or near some low-dimensional manifold embedded in the high dimensional space. For instance, natural signal variation among different subjects, fatigue, and delay in performing the tasks are very poorly approximated by changes in linear basic functions. However, previous studies suggest that manifold learning could capture these changes, and, using affine transformations, may even tolerate the effect of variations [54,55].

To better understand the suitability of this method for real-time decoding—for example to control a powered prosthesis—we needed to better understand the evolution of the classification accuracy over time. One pertinent question is how soon after touching the object would there be information in the muscle about the weight of the object that is decodable using this technique. For a running-window analysis, the shortest time-window possible using our technique (100 ms) suggested that the information exists in the muscle already within the first 100 ms after the subjects touch the object (Fig 9B). We also saw that the accuracy of our method was generally stable over the time duration when the subject grasped and moved the object. Achieving a stable decoding accuracy quickly after touching the object bodes well for the use of this technique in real time, though the relatively low accuracy over small time windows is a limitation worth noting. Therefore, constructing a combination of dimensionality reduction and classification techniques to specifically manage classification over small time windows is an interesting area of investigation for future studies.

## Conclusion, limitations and future work

This study proposes a complete, automated pipeline for the preprocessing, feature selection, feature extraction, and classification of objects of 3 different weights in a reach-grasp-lift task, where the only input was pre-processed EMG data from 5 muscles. Besides showcasing relatively high classification accuracy (F1 score 88.2±3.5%), our study highlights the importance of properly combining feature selection and classification algorithms to achieve this high accuracy.

The findings of our study are limited by a few factors. First, we used the open-source dataset, which has only 12 subjects, so the results of our statistical analyses should be interpreted cautiously. We have also left an analysis of the effect of fatigue on weight decoding for future studies. Nevertheless, given the high accuracy of our method overall, it is likely that the effect of fatigue on decoding accuracy is not dramatic. Similarly, the lower decoding accuracy of our method on smaller time windows deserves additional scrutiny.

## Supporting information

S1 Text Supplementary material. In this supplementary material, we give further details about the dimensionality-reduction methods we used.
(DOCX)
