# Supplementary material for: Dimensionality reduction for classification of object weight from electromyography
Source: PLoS One. 2021 Aug 16;16(8):e0255926. doi: 10.1371/journal.pone.0255926 (PMC8367006; doi:10.1371/journal.pone.0255926)
Supplement: S1 Text — In this supplementary material, we give further details about the dimensionality-reduction methods we used. (DOCX) [file pone.0255926.s001.docx]

Dimensionality Reduction for Classification of Object Weight from Electromyography

Supplementary Material

### Elnaz Lashgari and Uri Maoz

### In this supplementary material we give further details about the dimensionality-reduction methods we used. Much of this material follows Belkin et al. [1].

### Optimal embeddings

Given a data set, we construct a weighted graph G = (V, E) with edges connecting nearby points to each other (assuming the graph is connected). Consider the problem of mapping the weighted graph G to a line so that connected points stay as close together as possible. Let y = ${(y_{1}, y_{2}, \ldots, y_{n})}^{T}$ be such a map. A reasonable criterion for having good mapping is to minimize the following objective function:

| $\sum_{ij} {(y_{i}-y_{j})}^{2}W_{ij}$ | (1) |
| --- | --- |

under appropriate constraints. The objective function with our choice of weights $W_{ij}$ incurs a heavy penalty if neighboring points $x_{i}$ and $x_{j}$ are mapped far apart. Therefore, minimizing it is an attempt to ensure that if $x_{i}$ and $x_{j}$ are “close,” then $y_{i}$ and $y_{j}$ are close as well. It turns out that for any y, we have:

| $\frac{1}{2}\sum_{ij} \left( y_{i}-y_{j} \right)^{2}W_{ij}= y^{T}Ly$ | (2) |
| --- | --- |

Where as $L = D - W$ and $W_{ij}$ is symmetric and $D_{ii} =\sum_{j} W_{ji}$. Thus,

| $\sum_{ij} \left( y_{i}-y_{j} \right)^{2}W_{ij}= \sum_{ij} {{(y}_{i}}^{2}+{y_{j}}^{2}-2y_{i}y_{j}{)W}_{ij}=\sum_{i} {y_{i}}^{2}D_{ii}$+$\sum_{j} {y_{j}}^{2}D_{jj}$-2$\sum_{ij} y_{i}y_{j}W_{ij}=2y^{T}Ly$ | (3) |
| --- | --- |

Note that this calculation also shows that L is positive semidefinite. Therefore, the minimization problem reduces to finding: $\mathrm{argmin}y^{T}Ly s.t. y^{T}Dy=1$

The constraint $y^{T}Dy=1$ removes an arbitrary scaling factor in the embedding. Matrix D provides a natural measure on the vertices of the graph. The bigger the value $D_{ii}$ (corresponding to the $ith$ vertex) is, the more important is that vertex. Because L is positive semidefinite, the vector y that minimizes the objective function is given by the minimum eigenvalue solution to the generalized eigenvalue problem: $Ly = \lambda Dy$

Let **1** be the constant function taking 1 at each vertex. It is easy to see that **1** is an eigenvector with eigenvalue 0. If the graph is connected, **1** is the only eigenvector for λ = 0. To eliminate this trivial solution, which collapses all vertices of G onto the real number 1, we put an additional constraint of orthogonality and look for $\mathrm{argmin}y^{T}Ly s.t. y^{T}Dy=1$and $y^{T}D\boldsymbol{1}=0$. Thus, finally, the solution is now given by the eigenvector with the smallest nonzero eigenvalue. The condition $y^{T}D\mathbf{1}=0$can be interpreted as removing a translation invariance in y.

Now consider the more general problem of embedding the graph into an *m*-dimensional Euclidean space. The embedding is given by the $k\times m$ matrix $\Upsilon=[y_{1}, y_{2}, \ldots, y_{m} ],$ where the $i^{th}$ row provides the embedding coordinates of the $ith$ vertex. Similarly, we need to minimize:

| $\sum_{ij} \left\Vert y^{(i)}- y^{(j)} \right\Vert^{2}W_{ij}= {tr(\Upsilon}^{T}L\Upsilon)$ | (4) |
| --- | --- |

where $y^{(i)}=[{y_{1}\left( i \right), \ldots, y_{m}\left( i \right)]}^{T}$is the m-dimensional representation of the *i*^th^ vertex. This reduces to finding:

| $\mathrm{argmin} {tr(\Upsilon}^{T}L\Upsilon)$ s.t. $\Upsilon^{T}L\Upsilon=1$ | (5) |
| --- | --- |

For the one-dimensional embedding problem, the constraint prevents collapse onto a point. For the *m*-dimensional embedding problem, the constraint presented above prevents collapse onto a subspace of dimension less than $m - 1$.

The above therefore suggest that the Laplacian-Eigenmap algorithm keeps samples from the original, higher-dimensional space close to each other also in the lower-dimensional embedding. The Laplacian graph is analogous to the Laplace-Beltrami operator on manifolds. The eigenfunctions of the Laplace Beltrami operator have properties desirable for embedding [1, 2]. Let $\mathcal{M}_{x}\subseteq\mathbb{R}^{d}$ be a smooth, compact manifold embedded in a d-dimensional Euclidean space. A function ${f\mathcal{: M}}_{x}\to\mathbb{R}^{d}$ is said to be smooth if $f\in C^{\infty}\left( \mathcal{M}_{x}\mathbb{,R} \right)$, that is, the function $f$ and all of its derivatives are continuous. Let us define a different notion of smoothness related to the Laplace-Beltrami operator. The Laplace-Beltrami operator $L_{x}$ is a linear operator generalizing the Laplacian on Euclidean spaces to Riemannian manifolds. The eigenfunctions $\psi_{i}$ of the Laplace-Beltrami operator span a dense subset of the function space $H^{0}=L^{2}\left( \mathcal{M}_{x}\mathbb{,R} \right)$. The eigenvalues of the Laplace-Beltrami are real (and non-negative), so we can sort the associated eigenfunctions $\psi_{i}$ such that $\lambda_{i}\leq\lambda_{j}$for $i < j$. We say that $\psi_{i}$ is smoother than $\psi_{j}$ if $\lambda_{i}\leq\lambda_{j}$. It was shown that the best representation basis, in terms of truncated representation of functions ${f\mathcal{: M}}_{x}\to\mathbb{R}^{d}$ such that $\parallel\nabla f\parallel\leq1$, are in fact the eigenfunctions of the Laplace-Beltrami operator $L_{x}$. Thus, in that sense, we say that $f_{i}: \mathcal{M}_{x}\mathbb{\to R}$with $\parallel f_{i}\parallel= 1$ is smoother than $f_{j} : \mathcal{M}_{x}\mathbb{\to R}$ with $\parallel f_{j}\parallel= 1$ if $\parallel L_{x}f_{i}\parallel$ ≤ ∥$L_{x}f_{j}$ ∥.

Heat kernels and the choice of weight matrix: The Laplace Beltrami operator on differentiable functions on a manifold $\mathcal{M}$ is intimately related to heat flow. Let $f : M\mathbb{\to R}$ be the initial heat distribution and $u(x, t)$ be the heat distribution at time $t \left( u\left( x, 0 \right)= f \left( x \right) \right)$ (see [1, 2] for more details). The results show that we compute the graph Laplacian with the following weights:

| $W_{ij}=\left\{ \begin{aligned} e^{\frac{\left\Vert x_{i}-x_{j} \right\Vert^{2}}{4t} if \left\Vert x_{i}-x_{j} \right\Vert<\varepsilon} \\ 0 \mathrm{otherwise} \end{aligned} \right.$ | (6) |
| --- | --- |

### Properties of techniques for dimensionality reduction

In S1 Table 1, the dimensionality reduction techniques are listed by four general properties: (1) whether the mapping between the high-dimensional and the low-dimensional space is parametric, (2) the main free parameters to be optimized, (3) the computational complexity of the main computational part of the technique, and (4) the memory complexity of the technique [3, 4]. S1 Table 1 shows that most techniques for dimensionality reduction are non-parametric. This means that the technique does not specify a direct mapping from the high-dimensional to the low-dimensional space (or vice versa). The non-parametric nature of most techniques is a disadvantage for two main reasons: (1) it is not possible to generalize the mapping to a held-out or to a new test set (without carrying out the dimensionality-reduction technique again); (2) it is difficult to obtain insights into how much information of the high-dimensional data was preserved in the low-dimensional space by reconstructing the original data from its low-dimensional representation and measuring the error between the reconstructed and original data.

**S1 Table 1. Properties of techniques for dimensionality reduction**

| **Dimensionality reduction technique** | **Parametric** | **Free parameters** | **Computational complexity** | **Memory complexity** |
| --- | --- | --- | --- | --- |
| **PCA** | Yes | *none* | ${O(D}^{3})$ | ${O(D}^{2})$ |
| **ISOMAP** | No | *k* | ${O(n}^{3})$ | ${O(n}^{2})$ |
| **LLE** | No | *k* | ${O(pn}^{2})$ | ${O(pn}^{2})$ |
| **Laplacian Eigenmaps** | No | *k,* $\sigma$ | ${O(pn}^{2})$ | ${O(pn}^{2})$ |
| **t-SNE** | No | perplexity | ${O(n}^{2})$ | ${O(n}^{2})$ |

As for the free parameters, S1 Table 1 shows that the objective functions of most non-linear techniques for dimensionality reduction have free parameters that need to be optimized. In other words, there are parameters that directly influence the optimized cost function. Non-convex techniques for dimensionality reduction have additional free parameters, such as the learning rate and the permitted maximum number of iterations. Moreover, LLE uses a regularization parameter in the computation of the reconstruction weights.

The presence of free parameters has both advantages and disadvantages. The main advantage of free parameters is that they make the technique more flexible. However, they then need to be tuned to optimize performance. S1 Table 1 also provides more details on the computational and memory complexities of the techniques. The computational complexity of a dimensionality-reduction technique is important for its practical applicability. Algorithms grow increasingly infeasible as computational or memory demands rise. The computational complexity of a dimensionality reduction technique is determined by: (1) properties of the dataset, such as the number of datapoints *n* and their dimensionality *D*, and (2) by parameters of the techniques, such as the target dimensionality *d*, the number of nearest neighbors *k*, $\sigma$ (for techniques based on neighborhood graphs). In S1 Table 1, *p* denotes the ratio of nonzero elements in a sparse matrix to the total number of elements.

In the next section, we show how we deal with out-of-sample extension where there is no explicit projection function between the original data and their low dimensional representations in the original LE algorithm.

## **Out-of-sample extension**

An important requirement for dimensionality reduction techniques is the ability to embed new high-dimensional datapoints into an existing low-dimensional data representation. However, there is no explicit projection function between the original data and their low dimensional representations in the original LE algorithm, which makes out-of-sample extension difficult. To find projection of any additional samples, LE needs to be run on all the data together with the additional samples, resulting in considerable computational cost, especially when applying it to [large scale data](https://www.sciencedirect.com/topics/computer-science/large-scale-data) pattern recognition. Fortunately, various methods have been developed to mitigate the out-of-sample problem [5]: Linear approximation to LE, Kernel extensions to LE, Tensor representation of LE, incremental learning for LE, neural network approaches, and Extreme Learning Machine. The out-of-sample extension for spectral techniques has been presented in [6]. Nyström approximation supports out-of-sample extensions for spectral techniques such as ISOMAP, LLE, and Laplacian Eigenmaps. In the next section, we explain Nyström approximation in greater detail.

### Nyström extension

Let *D* denote the dimension of the initial set, *N* the number of samples (or points), $x_{i}$ a sample in $X\in\mathbb{R}^{D}$ and the $D \times N$ training matrix containing the samples. Let $y_{i}$ denote the coordinates in the embedded space, included in $\mathbb{R}^{d}$ where $d$ is the reduced dimension that corresponds to $x_{i}$. Finally, let $x_{N+1}$ denote a sample not belonging to the initial set of samples—i.e. an out-of-sample point. The goal is to estimate its reduced coordinates $y_{N+1}$. The Nyström method speeds up kernel-method computations by performing the eigen-decomposition on a subset of examples [7]. It was previously used to propose an out-of-sample extension to kernel-based spectral methods [6]. Let us recall the general framework in which spectral dimension-reduction techniques can be cast. Let $W$ be a symmetric matrix of size $N \times N$, expressing the affinity between the N points of the training set. Let $K(\cdot, \cdot)$ denote a data-dependant kernel function giving rise to matrix $W$ with $W_{ij}=K(x_{i},x_{j}).$

Let ($v_{k}$, $\lambda_{k}$) denote the eigenvector and eigenvalue pairs such that $Wv_{k} = \lambda_{k} v_{k}$. For dimensionality reduction, retain the $d$ largest (or smallest, depending on the method) eigenvalues and their associated eigenvectors. The embedding (or reduced coordinates) of each training sample $x_{i}$ is the ith row of a matrix $U$ that contains the *d* eigenvectors in columns. The Nyström extension for an out-of-sample point is a weighted sum of the previously calculated eigenvectors and eigenvalues. More precisely, the kth reduced coordinate of the out-of- sample point is approximated as: $y_{N+1}=\frac{1}{\lambda_{k}}\sum_{i=1}^{N} v_{ki}K\left( x_{N+1},x_{i} \right)$ for all $k=1, \ldots, d$ or, in matrix form: $\hat{y}_{N+1}=\frac{1}{\sqrt{\lambda}}U^{T}K_{N+1},$ where $\frac{1}{\sqrt{\lambda}}=diag (\frac{1}{\sqrt{\lambda_{1}}}, \ldots,\frac{1}{\sqrt{\lambda_{d}}})$. $U$ is the matrix whose columns are the eigenvectors, and $K_{N+1}=[K\left( x_{N+1},x_{1} \right)\ldots K\left( x_{N+1},X_{N} \right)]$. In [4], Bengio et al. have designed a formulation of $K (\cdot, \cdot)$ for Laplacian eigenmaps:

$K\left( a,b \right)=\frac{1}{n} \frac{K(a,b)}{\sqrt{E_{x}[K\left( a,x \right)]E_{x^{'}}[K\left( b,x^{'} \right)]}}$ (7)

The Nyström extension is applicable to any technique that make use of a kernel function. This method requires some parameter choice for the kernel $K (\cdot, \cdot)$, usually made heuristically.

**S1 Table 2. Statistical analysis Performance of different classifiers**

| **Paired Samples T-Test** | | | | | | | | | | | | | | | | | |
| --- | --- | --- | --- | --- | --- | --- | --- | --- | --- | --- | --- | --- | --- | --- | --- | --- | --- |
| **Classifiers** | |  | |  | | **t** | | **df** | | **p** | | **Mean Difference** | | **SE Difference** | | **Cohen's d** | |
| k-NN |  | - |  | RBF SVM |  | 4.000 |  | 6 |  | 0.007 |  | 6.771 |  | 1.693 |  | 1.512 |  |
| k-NN |  | - |  | Linear SVM |  | 5.616 |  | 6 |  | 0.001 |  | 17.257 |  | 3.073 |  | 2.123 |  |
| k-NN |  | - |  | Random Forest |  | 2.928 |  | 6 |  | 0.026 |  | 5.671 |  | 1.937 |  | 1.107 |  |
|  | | | | | | | | | | | | | | | | | |
| *Note.*  Student's t-test. | | | | | | | | | | | | | | | | | |

## **S1 Table 3. Statistical analysis Performance of different dimension reduction techniques**

| **Paired Samples T-Test** | | | | | | | | | | | | | |
| --- | --- | --- | --- | --- | --- | --- | --- | --- | --- | --- | --- | --- | --- |
|  | |  | |  | | **t** | | **df** | | **p** | | **Cohen's d** | |
| LE (simple minded) |  | - |  | PCA |  | -5.613 |  | 11 |  | < .001 |  | -1.620 |  |
| LE (simple minded) |  | - |  | ISOMAP |  | -3.721 |  | 11 |  | 0.003 |  | -1.074 |  |
| LE (simple minded) |  | - |  | LLE |  | 2.214 |  | 11 |  | 0.049 |  | 0.639 |  |
| LE (simple minded) |  | - |  | LE (rbf) |  | 1.064 |  | 11 |  | 0.310 |  | 0.307 |  |
| LE (simple minded) |  | - |  | t-SNE |  | -7.844 |  | 11 |  | < .001 |  | -2.264 |  |
| LE (rbf) |  | - |  | PCA |  | -5.158 |  | 11 |  | < .001 |  | -1.489 |  |
| LE (rbf) |  | - |  | ISOMAP |  | -3.882 |  | 11 |  | 0.003 |  | -1.121 |  |
| LE (rbf) |  | - |  | LLE |  | 1.161 |  | 11 |  | 0.270 |  | 0.335 |  |
| LE (rbf) |  | - |  | t-SNE |  | -6.148 |  | 11 |  | < .001 |  | -1.775 |  |
|  | | | | | | | | | | | | | |
| Note.  Student's t-test. | | | | | | | | | | | | | |

## **S1 Table 4. Repeated Measures ANOVA**

| Within Subjects Effects | | | | | | | | | | | | | | |
| --- | --- | --- | --- | --- | --- | --- | --- | --- | --- | --- | --- | --- | --- | --- |
|  | | | **Sum of Squares** | | **df** | | **Mean Square** | | | | **F** | | **p** | |
| Classifiers | |  | 1087.127 |  | 3 |  | 362.376 | | |  | 15.490 |  | < .001 |  |
| Residual | |  | 421.096 |  | 18 |  | 23.394 | | |  |  |  |  |  |
|  | | | | | | | | | | | | | | |
| Note.  Type III Sum of Squares | | | | | | | | | | | | | | |
| Between Subjects Effects | | | | | | | | | | | |  |  |  |
|  | | **Sum of Squares** | | **df** | | **Mean Square** | | **F** | | **p** | |  |  |  |
| Residual |  | 125.647 |  | 6 |  | 20.941 |  |  |  |  |  |  |  |  |
|  | | | | | | | | | | | |  |  |  |
| Note.  Type III Sum of Squares | | | | | | | | | | | |  |  |  |

| **S1 Table 5. Post Hoc Comparisons - Classifiers** | | | | | | | | | | | | | |
| --- | --- | --- | --- | --- | --- | --- | --- | --- | --- | --- | --- | --- | --- |
|  | |  | | **Mean Difference** | | **SE** | | **t** | | **Cohen's d** | | **p _holm_** | |
| Linear SVM |  | RBF SVM |  | -10.486 |  | 3.170 |  | -3.307 |  | -1.250 |  | 0.049 |  |
|  |  | Random Forest |  | -11.586 |  | 2.703 |  | -4.286 |  | -1.620 |  | 0.026 |  |
|  |  | k-NN |  | -17.257 |  | 3.073 |  | -5.616 |  | -2.123 |  | 0.008 |  |
| RBF SVM |  | Random Forest |  | -1.100 |  | 2.586 |  | -0.425 |  | -0.161 |  | 0.685 |  |
|  |  | k-NN |  | -6.771 |  | 1.693 |  | -4.000 |  | -1.512 |  | 0.028 |  |
| Random Forest |  | k-NN |  | -5.671 |  | 1.937 |  | -2.928 |  | -1.107 |  | 0.053 |  |
|  | | | | | | | | | | | | | |
| *Note.*  Cohen's d does not correct for multiple comparisons. | | | | | | | | | | | | | |
| *Note.*  Bonferroni adjusted confidence intervals. | | | | | | | | | | | | | |

## **References**

1. Belkin, M. and P. Niyogi, *Laplacian eigenmaps for dimensionality reduction and data representation.* Neural computation, 2003. **15**(6): p. 1373-1396.

2. Yair, O., et al., *Spectral Discovery of Jointly Smooth Features for Multimodal Data.* arXiv preprint arXiv:2004.04386, 2020.

3. Van Der Maaten, L., E. Postma, and J. Van den Herik, *Dimensionality reduction: a comparative.* J Mach Learn Res, 2009. **10**(66-71): p. 13.

4. Van der Maaten, L., E.O. Postma, and H.J. van den Herik, *Matlab toolbox for dimensionality reduction.* MICC, Maastricht University, 2007.

5. Li, B., Y.-R. Li, and X.-L. Zhang, *A survey on Laplacian eigenmaps based manifold learning methods.* Neurocomputing, 2019. **335**: p. 336-351.

6. Bengio, Y., et al. *Out-of-sample extensions for lle, isomap, mds, eigenmaps, and spectral clustering*. in *Advances in neural information processing systems*. 2004.

7. Quispe, A.M., C. Petitjean, and L. Heutte, *Extreme learning machine for out-of-sample extension in Laplacian eigenmaps.* Pattern Recognition Letters, 2016. **74**: p. 68-73.
